# Pembrolizumab and Olaparib (POLAR) Maintenance Therapy in Metastatic Pancreatic Cancer With or Without Homologous Repair Deficiency: A Biomarker Selected Phase II Trial

**DOI:** 10.21203/rs.3.rs-7334701/v1

**Published:** 2025-09-01

**Authors:** Wungki Park, Catherine O’Connor, Joanne Chou, Marc Hilmi, Zeynep Tarcan, Carly Schwartz, Mary Larsen, Walid Chatila, Karthigayini Sivaprakasam, Shigeaki Umeda, Maria Perry, Anna Varghese, Kenneth Yu, Fiyinfolu Balogun, Alice Zervoudakis, Seth Katz, Tae-Hyung Kim, Ken Zhao, Allison Richards, Nicolas Lecomte, Daniel Muldoon, Elias Karnoub, Jessica Yang, Imane El-Dika, Devika Rao, Joshi Smita, Michael Foote, Ryan Sugarman, James Harding, Andrew Epstein, David Kelsen, Sree Chalasani, Fergus Keane, Joshua Schoenfeld, Anupriya Singhal, Erin Diguglielmo, Chaitanya Bandlamudi, Junmin Song, Hulya Sahin Ozkan, Jungeui Hong, Haochen Zhang, Agustin Cardenas, Maria Lao, Jerry Melchor, Ronak Shah, Wenfei Kang, Francesca Mazzoni, Kevin Soares, Mark Donoghue, Vinod Balachandran, Mark Schattner, Ernesto Santos, Vineet Rolston, Marsha Reyngold, Alice Wei, Ramzi Homsi, Murray Tipping, Olca Basturk, Michael Berger, Richard Do, William Jarnagin, Nadeem Riaz, Dana Pe’er, Marinela Capanu, Christine Iacobuzio-Donahue, Eileen O’Reilly

**Affiliations:** Memorial Sloan Kettering Cancer Center; Harvard Medical School, Boston, MA, USA, David M. Rubenstein Center for Pancreas Cancer Research, Memorial Sloan Kettering Cancer Center; Memorial Sloan Kettering Cancer Center; MSKCC; Human Oncology and Pathogenesis Program, David M. Rubenstein Center for Pancreas Cancer Research, Memorial Sloan Kettering Cancer Center; Department of Medicine, David M. Rubenstein Center for Pancreas Cancer Research; Department of Medicine, David M. Rubenstein Center for Pancreas Cancer Research; Memorial Sloan Kettering Cancer Center; Department of Pathology and Laboratory Medicine, Marie-Josée and Henry R. Kravis Center for Molecular Oncology, Memorial Sloan Kettering Cancer Center; Memorial Sloan Kettering Cancer Center; Memorial Sloan Kettering Cancer Center; Memorial Sloan Kettering Cancer Center; Memorial Sloan Kettering Cancer Center; Memorial Sloan Kettering Cancer Center; Department of Medicine, David M. Rubenstein Center for Pancreas Cancer Research, Weill Cornell Medical College, Memorial Sloan Kettering Cancer Center; Department of Radiology, David M. Rubenstein Center for Pancreas Cancer Research, Memorial Sloan Kettering Cancer Center; Department of Radiology, David M. Rubenstein Center for Pancreas Cancer Research, Memorial Sloan Kettering Cancer Center; Department of Radiology, Memorial Sloan Kettering Cancer Center; Memorial Sloan Kettering Cancer Center; Memorial Sloan Kettering Cancer Center; Memorial Sloan Kettering Cancer Center; Human Oncology and Pathogenesis Program, David M. Rubenstein Center for Pancreas Cancer Research, Memorial Sloan Kettering Cancer Center; Department of Medicine, Weill Cornell Medical College, Memorial Sloan Kettering Cancer Center; Department of Medicine, Weill Cornell Medical College, Memorial Sloan Kettering Cancer Center; Department of Medicine, Weill Cornell Medical College, Memorial Sloan Kettering Cancer Center; Department of Medicine, Weill Cornell Medical College, Memorial Sloan Kettering Cancer Center; Memorial Sloan Kettering Cancer Center; Department of Medicine, Weill Cornell Medical College, Memorial Sloan Kettering Cancer Center; Department of Medicine, Weill Cornell Medical College, Memorial Sloan Kettering Cancer Center; Memorial Sloan Kettering Cancer Center; Memorial Sloan Kettering Cancer Center; Department of Medicine, Weill Cornell Medical College, Memorial Sloan Kettering Cancer Center; Department of Medicine, David M. Rubenstein Center for Pancreas Cancer Research; Department of Medicine, David M. Rubenstein Center for Pancreas Cancer Research, Weill Cornell Medical College, Memorial Sloan Kettering Cancer Center; Memorial Sloan Kettering Cancer Center; Department of Medicine, David M. Rubenstein Center for Pancreas Cancer Research; Memorial Sloan Kettering Cancer Center; Department of Medicine, David M. Rubenstein Center for Pancreas Cancer Research, Memorial Sloan Kettering Cancer Center; Human Oncology and Pathogenesis Program, David M. Rubenstein Center for Pancreas Cancer Research, Memorial Sloan Kettering Cancer Center; Memorial Sloan Kettering Cancer Center; Valar Labs; Human Oncology and Pathogenesis Program, David M. Rubenstein Center for Pancreas Cancer Research, Memorial Sloan Kettering Cancer Center; Human Oncology and Pathogenesis Program, David M. Rubenstein Center for Pancreas Cancer Research, Memorial Sloan Kettering Cancer Center; Memorial Sloan Kettering Cancer Center; Department of Pathology and Laboratory Medicine, Marie-Josée and Henry R. Kravis Center for Molecular Oncology, Memorial Sloan Kettering Cancer Center; Molecular Cytology Core Facility, Memorial Sloan Kettering Cancer Center; Molecular Cytology Core Facility, Memorial Sloan Kettering Cancer Center; Department of Surgery, Immuno-Oncology Program, The Olayan Center for Cancer Vaccines, Memorial Sloan Kettering Cancer Center; Memorial Sloan Kettering Cancer Center; Memorial Sloan Kettering Cancer Center; Memorial Sloan Kettering Cancer Center; Department of Radiology, Memorial Sloan Kettering Cancer Center; Department of Medicine, David M. Rubenstein Center for Pancreas Cancer Research, Weill Cornell Medical College, Memorial Sloan Kettering Cancer Center; Department of Radiology, David M. Rubenstein Center for Pancreas Cancer Research, Weill Cornell Medical College, Memorial Sloan Kettering Cancer Center; Memorial Sloan Kettering; Department of Pathology and Laboratory Medicine, Marie-Josée and Henry R. Kravis Center for Molecular Oncology, Memorial Sloan Kettering Cancer Center; Molecular Cytology Core Facility, Memorial Sloan Kettering Cancer Center; Memorial Sloan Kettering Cancer Center; Memorial Sloan Kettering Cancer Center; Memorial Sloan Kettering Cancer Center; Memorial Sloan-Kettering Cancer Center; Memorial Sloan Kettering Cancer Center; Memorial Sloan Kettering Cancer Center; Memorial Sloan Kettering Cancer Center

## Abstract

The phase 2 POLAR trial evaluated maintenance pembrolizumab plus olaparib in 63 participants with metastatic pancreatic cancer with disease control on platinum-based chemotherapy. Participants were prospectively stratified into three cohorts by type of HRD: Cohort A (homologous recombination deficient [HRD] by *BRCA1/2* or *PALB2* mutations, N=33), Cohort B (non-core HRD mutations, N=15), and Cohort C (platinum-sensitive without HRD mutations, N=15). Cohort A used a two-stage design with co-primary endpoints of objective response rate (ORR) ≥43% and 6-month progression-free survival ≥77% per Response Evaluation Criteria in Solid Tumors (RECIST) version 1.1. For Cohort A, ORR was 35% (95% CI: 15–59) and 6-month-PFS rate was 64% (95% CI: 49–82), not meeting the primary endpoint. Among surviving participants (N=17), the median follow-up was 26.0 months (range: 1.4–52.5), and the 2-year overall survival rate was 56% (95% CI: 41–76). Median PFS for Cohort A was 8.3 months (95% CI: 5.3-not reached), 4.8 months (95% CI: 4–12) for Cohort B, and 3.3 months (95% CI: 1.9–4.8) for Cohort C. Pre-planned translational profiling demonstrated that molecular response by circulating cell-free DNA (cfDNA), high tumor-infiltrating lymphocyte (TIL) density, and increased abundance of frameshift indels and neoantigens were associated with durable benefit, particularly in HRD tumors. These findings support a precision immunotherapy approach for biomarker-defined subsets of pancreatic cancer. ClinicalTrials.gov identifier: NCT04666740

## Introduction

Pancreatic cancer (PC) is projected to become the second leading cause of cancer-related deaths by 2030^1,2^. Despite therapeutic advancements, only modest survival improvements have been achieved with multi-agent chemotherapy regimens, and genomically unselected PC demonstrates near-universal resistance to immune checkpoint blockades (ICB). In light of the challenges with long-term chemotherapy tolerance, de-escalation and maintenance strategies have gained traction as a means to improve quality of life while maintaining disease control^3,4^. Notably, olaparib, a poly-(ADP-ribose) polymerase inhibitor (PARPi), became the first targeted maintenance therapy to demonstrate clinical benefit in genetically selected patients with homologous recombination deficient (HRD) PC as defined by germline *BRCA1/2* mutations^5,6^.

Precision cancer therapy in biomarker-selected subsets is feasible in PC and has improved survival outcomes when participants receive matched targeted therapies^7,8^. A rare (1%) subset with mismatch repair deficient (dMMR) PC, defined by a hypermutable phenotype with a higher mutation-derived neoantigen burden, benefit from ICB targeting programmed-death 1 (PD-1)^9,10^. Similarly, a significant clinical benefit from platinum-based therapy has been demonstrated in patients with PC harboring a core HRD mutation (<9%), which includes germline or somatic *BRCA1*, *BRCA2*, and *PALB2* mutations. PARPi, as monotherapy and in combination with chemotherapy has been investigated for core as well as non-core HRD (harboring other candidate HRD gene mutations)^5,11,12^. In parallel, early-phase studies targeting *KRAS* mutations, present in over 90% of PC, have reported encouraging safety profiles and early signs of clinical activity^13,14^. Finally, in a select subset of resected PC, neoantigen-targeted vaccines against personalized or shared neoantigens, have demonstrated encouraging safety and early signs of clinical activity, further highlighting the potential of immune-mediated disease control in genomically defined subsets of PC^15–17^.

Over the past decade, large-scale sequencing efforts have revealed that genomic instability in HRD tumors is associated with characteristic genomic signatures, including the COSMIC signature 3, and large-scale structural changes^18–20^. These structural changes include large-scale state transitions (LST), loss of heterozygosity (LOH), and telomeric allelic imbalance (TAI), and tumors with elevated number of these events have been shown to be more sensitive to platinum-based chemotherapy and PARPi^19,21,22^. Interestingly, some of these mutational patterns, such as increased frameshift indels and specific genomic signatures, particularly in tumors with *BRCA2* mutations, may contribute to increased immunogenicity^19,23,24^. Lastly, emerging data suggest that mutations in non-core HRD genes (e.g., *RAD51B/C/D*, *ATM, CHEK2, BAP1, BARD1, BRIP1*) may confer HRD-like biology and warrant further clinical and mechanistic evaluation^19,22,25^.

The POLAR trial is a phase 2, single-center, non-randomized biomarker-selected basket study evaluating the combination of pembrolizumab (anti-PD-1) and olaparib (PARP) inhibitors as maintenance therapy in participants with metastatic PC who achieved disease control after platinum-based chemotherapy (NCT04666740)^26^. Given the known clinical benefit of PARPi in core HRD tumors and the investigational predictive value of non-core HRD mutations or platinum sensitivity alone, participants were prospectively stratified into three cohorts: (A) HRD by *BRCA1/2* or *PALB2* mutations, (B) non-core HRD (ncHRD) gene mutations, and (C) platinum-sensitive without HRD mutations. Herein, we report clinical outcomes across all three cohorts, along with translational analyses, including mutational signatures, neoantigen, and immune correlates to identify biological features associated with therapeutic response.

## Results

### Study Design

**Figure 1.**
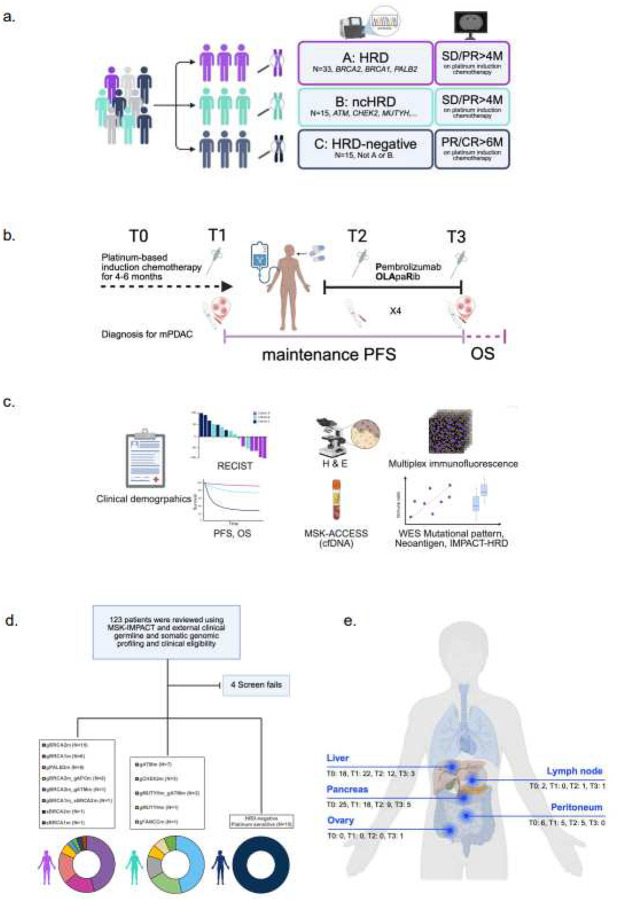
Schema of the POLAR trial: Precision immunotherapy for genetically and phenotypically defined subsets of metastatic pancreatic cancer. (a) Cohort stratification. Participants with metastatic pancreatic cancer (mPC) were prospectively assigned to one of three cohorts based on germline or somatic mutation profiling and clinical response to platinum-based chemotherapy: Cohort A (HRD, N=33): Core HRR gene mutations (*BRCA1*, *BRCA2*, *PALB2*). Cohort B (ncHRD, N=15): Non-core HRR mutations (e.g., *ATM*, *CHEK2*, *MUTYH*). Cohort C (HRD-negative, N=15): No HRD-associated mutations but ≥6 months of platinum sensitivity. (b) Trial timeline. Schematic overview of treatment course: platinum-based induction chemotherapy (T0–T1), maintenance with pembrolizumab and olaparib, with progression-free survival (PFS: T1–T3), on-treatment biopsy (T2) and overall survival (OS) tracked throughout. (c) Correlative endpoints. Integrated clinical and translational analyses included demographics, RECIST v1.1 response, PFS/OS, cfDNA profiling (MSK-ACCESS), whole-exome sequencing (WES) for mutational patterns and neoantigens, and tumor microenvironment assessment by H&E and multiplex immunofluorescence (mIF). (d) Screening and cohort allocation. Among 123 patients screened using MSK-IMPACT and external germline/somatic testing, 63 were enrolled and assigned to each cohort. Bottom: Genomic breakdown by gene for each cohort. (e) Biospecimen collection sites across timepoints. Anatomical distribution of metastatic biopsies collected longitudinally (T0–T3), with most samples from liver, pancreas, and peritoneum. **Abbreviations:** HRD, homologous recombination deficiency; ncHRD, non-core HRD; mPC, metastatic pancreatic cancer; SD, stable disease; PR, partial response; CR, complete response; PFS, progression-free survival; OS, overall survival; RECIST, Response Evaluation Criteria in Solid Tumors; cfDNA, cell-free DNA; WES, whole-exome sequencing; mIF, multiplex immunofluorescence.

The POLAR trial enrolled N=63 participants into three cohorts based on molecular classification and response to platinum-based chemotherapy. Cohort A included patients with pathogenic germline or somatic mutations in HRD genes (*BRCA2, BRCA1, PALB2*), without progressive disease following over 4 months of platinum-based chemotherapy. Cohort B (ncHRD) included patients with pathogenic germline or somatic mutations in non-core HRD genes (*ATM, BAP1, BARD1, BLM, BRIP1, CHEK2, FAM175A, FANCA, FANCC, NBN, RAD50, RAD51, RTEL1, MUTYH*), also without progressive disease over 4 months of platinum-based chemotherapy, and Cohort C included patients with platinum-sensitivity for at least 6 months (12 cycles of modified FOLFIRINOX) but without HRD or ncHRD gene mutations (Fig. 1a).

All participants received maintenance olaparib 300 mg orally twice a day daily and pembrolizumab intravenously every 3 weeks for 6 months, and then every 6 weeks until disease progression or unacceptable toxicity intravenously. Select patients were allowed to continue beyond radiographic progression if deriving clinical benefit.

Serial tumor tissue (baseline, on-treatment, and post-progression when feasible) and matched peripheral blood samples were collected (Fig. 1b–d). These were used to investigate genomic instability, tumor–immune microenvironment, and neoantigen landscape features associated with therapeutic response and resistance. Specifically, whole exome sequencing (WES), cell-free DNA, and multiplex immunofluorescence were employed from available biospecimens collected from different sites at different timepoints (Fig. 1c,e), to evaluate mutational signatures, neoantigens, and immune infiltration across responders and non-responders (*see* Methods). This translational framework enabled broad exploratory analyses of biological correlates of response to POLAR.

### Baseline Characteristics

Baseline clinical characteristics are summarized in **Table 1**. The median age at diagnosis was numerically similar across cohorts (Median age of 62–65 years), with balanced sex distribution. Most participants presented with *de novo* stage IV disease, and all had an ECOG performance status of 0 or 1. Tumor histology was predominantly adenocarcinoma, with a small number (N=3) of acinar and adenosquamous carcinomas in Cohort A. Baseline CA19–9 and CEA values at enrollment were similar between cohort A and B but higher in Cohort C, consistent with greater disease burden. Molecular characteristics at enrollment of the 63 participants are summarized in Fig 1d **and Table 2**. Among Cohort A, *BRCA2* mutations were most common (N=18, 53%), followed by *BRCA1* (N=10) and *PALB2* (N=6). In Cohort B, *ATM* mutations were most common (N=8, 53%), followed by *CHEK2* mutations in N=3 (20%), *BLM* (N=1), *FANCC* (N=1), *MUTYH* (N=1), and *MUTYH* and *ATM* (N=1) co-mutated. Cohort C had no detectable HRD or ncHRD mutations by design.

### Primary Outcomes

**Figure 2.**
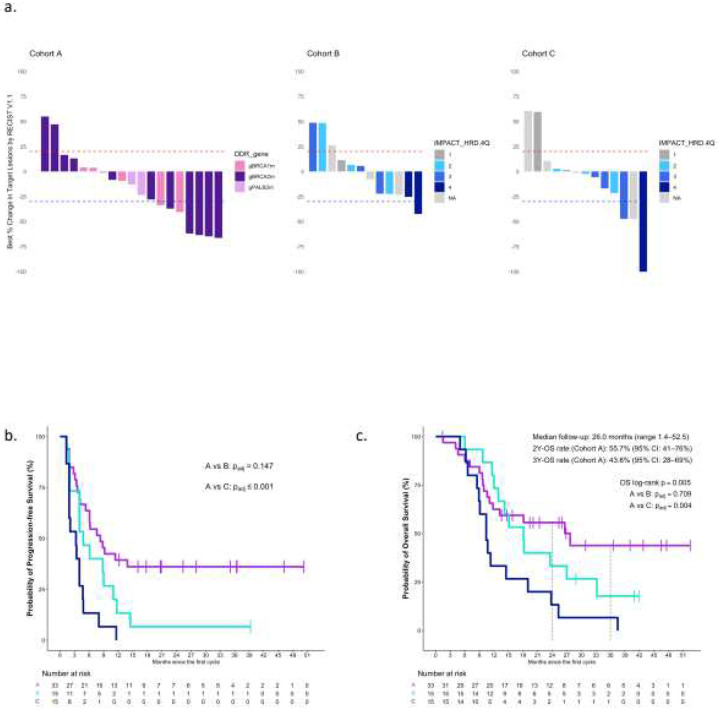
Clinical and molecular outcomes in the POLAR trial **a. Waterfall plots** show the best percent change in target lesion size per RECIST v1.1 in Cohorts A, B, and C. In Cohort A, bars are color-coded by the gene harboring a core HRD mutation: *BRCA1*, *BRCA2*, or *PALB2*. In Cohorts B and C, bars are shaded by IMPACT_HRD quartile, a genomic scar score derived from MSK-IMPACT targeted sequencing. Higher quartiles (3–4, darker blue) reflect greater levels of genomic instability associated with HRD and were used to stratify non-core HRD (B) and HRD-negative (C) tumors. **b. Kaplan–Meier curves** for progression-free survival (PFS) by cohort. Median PFS was 8.3 months in Cohort A, 4.8 months in Cohort B, and 3.3 months in Cohort C. Post-hoc pairwise comparisons showed a significant difference between Cohort A and C (*p*adj ≤ 0.001), but not between A and B (*p*adj = 0.147). **c. Kaplan–Meier curves** for overall survival (OS) by cohort. Median follow-up was 26.0 months (range, 1.4–52.5). In Cohort A, the 2-year OS rate was 56% (95% CI: 41–76%) and the 3-year OS rate was 44% (95% CI: 28–69%). OS was significantly improved in Cohort A compared to C (*p*adj = 0.004), with no difference between A and B (*p*adj = 0.709). Dashed lines indicate 24- and 36-month landmarks. Number at risk is shown below each plot. **Abbreviations:** PFS, progression-free survival; OS, overall survival; HRD, homologous recombination deficiency; RECIST, Response Evaluation Criteria in Solid Tumors v1.1; MSK-IMPACT, Memorial Sloan Kettering–Integrated Mutation Profiling of Actionable Cancer Targets.

Among 63 participants enrolled in the POLAR trial, 46 (73%) had RECIST-evaluable disease at initiation of maintenance therapy, as assessed by independent radiology review. Objective responses were observed across all three cohorts, though the number of responses varied by genomic context (Fig 2a). In Cohort A, the ORR was 35% (7 of 20; 95% CI: 15–59), not meeting the co-primary endpoint of ORR at 43%. In Cohorts B and C, the ORR was 8% (1 of 12; 95% CI: 0–38) and 14% (2 of 14; 95% CI: 2–43), respectively.

Progression-free survival (PFS) for each of the 3 cohorts is presented in Fig. 2b. At the time of data cutoff (June 25, 2025), the 6-month progression-free survival (PFS) rate was highest in Cohort A at 64% (95% CI: 49–82); however, it did not meet the co-primary endpoint of 6-month PFS rate of 77% (The protocol is attached as Supplementary 1). This was followed by Cohort B at 47% (95% CI: 27–80) and Cohort C at 13% (95% CI: 3.7–48). Median PFS was 8.3 months (95% CI: 5.3-NR), 4.8 months (95% CI: 4–12), and 3.3 months (95% CI: 1.9–4.8), separately for Cohort A, B and C. Post- hoc analyses showed the PFS distributions were significantly different between the 3 cohorts (p=<0.001): Cohort A vs B (p_adj_=0.147) and Cohort A vs Cohort C (p_adj_=<0.001).

### Secondary Outcomes

Disease control was achieved in the majority of participants, with disease control rates (DCR) of 80% in Cohort A (16 of 20; 95% CI: 56–94), 75% in Cohort B (9 of 12; 95% CI: 43–95), and 50% in Cohort C (7 of 14; 95% CI: 23–77). These findings mirrored the trend observed across PFS and OS, supporting the clinical relevance of genomic stratification. Among surviving patients from all 3 cohorts combined (N= 20), the median follow-up was 27.9 months (range: 1.4–52.5). Median overall survival (OS) for Cohort A was 28 months (95% CI: 12- NR), 18 months (95% CI: 13- NR) for Cohort B, followed by 10 months (95% CI: 8.9–24) for Cohort C (Fig 2). In Cohort A (Fig 2c), the 2-year OS rate was 56% (95% CI: 41–76) and 3-year OS rate was 44% (95% CI: 28–69). Post-hoc analyses showed significant difference in OS distributions between the 3 cohorts (p=0.005): Cohort A vs B (p_adj_=0.709) and Cohort A vs C (p_adj_=0.004)

### Subgroup Analysis by *BRCA2*, *PALB2*, *BRCA1*, and ncHRD Gene Mutations

Subgroup analysis by mutation type within Cohort A (HRD) and B (ncHRD) revealed heterogeneity in clinical outcomes. Participants with *BRCA2* (N=18) and *PALB2* mutations (N=6) demonstrated numerically similar PFS and OS, but longer than participants with *BRCA1* mutations (N=9) (**Supplementary Fig. 2**). The median PFS was 9.9 months (95% CI: 3.6-NR), 12.0 months (95% CI: 6.2-NR), and 6.1 months (95% CI: 4.1-NR), respectively for *BRCA2, PALB2*, and *BRCA1*. The median OS was 28.0 months (95% CI: 9.9-NR), 27.0 months (95% CI: 11-NR), and 18.0 months (95% CI: 12-NR), respectively. At 24 months, OS rates were 59% for *BRCA2*, 67% for *PALB2*, and 42% for *BRCA1*. From Cohort A (N=33), 15 participants had both germline and somatic sequencing data available, and the rest (N=18) had only cfDNA available. For zygosity status: 10 biallelic loss (7 *BRCA2*, 2 *PALB2*, and 1 *BRCA1*), 3 monoallelic loss (2 *BRCA2* and 1 *PALB2) and* 2 indeterminate (1 *BRCA2* and 1 *BRCA1)* (**Supplementary Tab. 1**). In Cohort B, *ATM* mutations were the most common type (N=9, 60%). Participants with *ATM* and non-*ATM* ncHRD gene mutations (*CHEK2, FANCC*, and *MUTYH*) had similar median PFS to other ncHRD mutations (4.8 months [95% CI: 2-NR] vs 6.5 months [95% CI: 4-NR]) (**Supplementary Fig. 3**). Participants with *ATM* mutation had numerically longer OS (18 months [95% CI: 15-NR] vs 14 months [95% CI: 12-NR]).

### Safety

Treatment was generally well tolerated in line with the known adverse event profiles of both drugs and no new safety signals were observed. No grade 4 or 5 treatment-related adverse events (TRAE) occurred. Grade 3 TRAE included anemia (N=10, 15%) and abdominal infection (N=1, 1.6%). Grade 2 immune-related adverse events (irAEs) included colitis (N=1), hyperglycemia (N=1), pneumonitis (N=2), pancreatitis (N=1), and hyperthyroidism (N=1). Grade 3 irAEs occurred in N=4 participants and included pneumonitis and colitis (**Table 3**).

### Exploratory Integrated Biomarker Analyses

#### cfDNA Dynamics and Clinical Benefit

To explore the association between early cell-free DNA (cfDNA) dynamics and clinical outcomes, we analyzed 30 representative participants’ plasma pairs (N=60) from baseline (T1) and 6-week (T2) plasma across the three cohorts (A: N=14 pairs, B: N=8 pairs, C: N=8 pairs) using MSK-ACCESS. Of these, N=56 samples (93%) passed quality control, and only N=32 (57%) had detectable somatic mutations. Notably, most samples at T1 had low mean variant allele frequency (VAF) less than 0.004 (**Supplementary Fig. 4a**), indicative of molecular residual disease (mRD). Change in mean VAF between T1 and T2 were generally minimal (range: −0.0031 – 0.013) and a trend toward higher VAF increases was observed among participants with progression-free survival (PFS) ≤6 months compared to those with PFS >6 months (p=0.063, **Supplementary Fig. 4b–c**). At data cutoff, 5 participants (7.9%) achieved durable clinical benefit with PFS exceeding 36 months. Four of these had undetectable or near-undetectable mean VAF (0.00014 and 0.00009) at both T1 and T2 timepoints; the T1 sample of the fifth participant did not pass quality control but T2 mean VAF was 0 (**Supplementary Fig. 4d**).

#### HRD Genotype and Immune-Related Adverse Events in Relation to Durable Benefit

**Figure 3.**
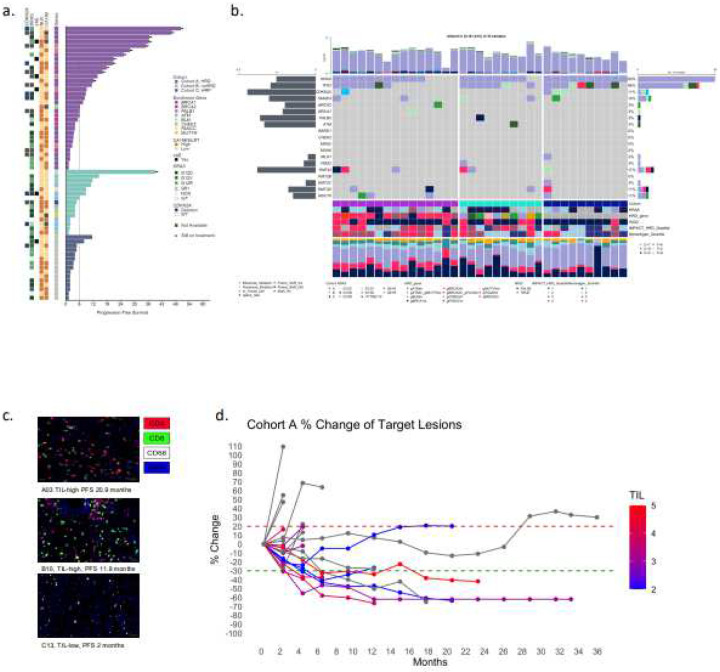
Clinical, genomic, and immunologic parameters associated with response to pembrolizumab and olaparib in POLAR trial. **a. Swimmer plot** showing PFS annotated with clinical and genomic features progression-free survival (PFS) for all POLAR participants, stratified by each cohort. Bars are annotated by enrollment gene (e.g., *BRCA1*, *BRCA2*, *PALB2*, *ATM*, *CHEK2*), baseline CA19–9 level, irAE status, and *KRAS* allele, and *CDKN2A* homozygous deletion status. **b. Oncoprint with whole exome sequencing (WES)** showing genomic mutations across POLAR patients (N=35) with annotation tracks for cohort, IMPACT-HRD scores, HRD gene class and neoantigen quartile. **c. Representative multiplex immunofluorescence images** from tumors with high (B10) and low (C13) T-cell infiltration. Tumors were stained for CD3 (red), CD8 (green), CD68 (white), and DAPI (blue). **d. Spaghetti plot:** Longitudinal changes of tumor response in patients with different levels of TIL in tumors of the participants from Cohort A. Lines colored by TIL density score. TIL-high tumors were associated with longer PFS. **Abbreviations:** HRD, homologous recombination deficiency; ncHRD, non-core HRD; HRP, homologous recombination proficient; GIS, genomic instability score; TMB, tumor mutational burden; TIL, tumor-infiltrating lymphocyte; PFS, progression-free survival; RECIST, Response Evaluation Criteria in Solid Tumors; irAE, immune-related adverse event.

To explore the relationship between clinical and molecular features associated with clinical benefit, we generated participant-level Swimmer plots (Fig. 3a) integrating PFS, genomic mutations (HRD and ncHRD genes, *KRAS*, and *CDKN2A* homozygous deletion), baseline biomarkers (CA19–9 and neutrophil-to-lymphocyte ratio [NLR]), and immune-related adverse events (irAEs). Eleven durable responses were predominantly observed in Cohort A, including exceptionally long-term responders (PFS > 18 months) with *BRCA2* (N=8), *PALB2* (N=2), and *BRCA1* (N=1) germline mutations. One participant in Cohort B with a germline *BLM* mutation has remained on POLAR for more than 36 months with a durable partial response (−42.2% by RECIST v1.1). All irAEs (N=6) occurred among patients who had PFS > 6 months across three cohorts. Other variables such as *KRAS* mutation subtype, CDKN2A loss, and baseline NLR did not separate groups with different PFS outcome.

#### Cohort A (HRD) Tumors are Enriched for Immunogenic Mutational Patterns and Immune Infiltration

Whole-exome sequencing (WES) of 35 baseline tumors (N=15, N=10 and N=10 in Cohort A, B, and C) revealed that Cohort A (HRD) exhibited a distinct and significantly more immunogenic mutational landscape compared to Cohort C (platinum-sensitive without HRD) (**Supplementary Fig. 5**). Specifically, both total indel burden (median of 10 [IQR 5–12]) and frameshift indel burden (median: 8 [IQR: 4–11]) were significantly higher in Cohort A than Cohort C (median of 2 for both parameters) (p<0.01), indicating active error-prone repair processes that preferentially generate mutation-derived neoantigens (**Supplementary Fig. 5b**). While non-synonymous single nucleotide variant (nsSNV) burden and total predicted neoantigen burden did not different significantly across cohorts, this enrichment of frameshift mutations in HRD tumors likely contributes to enhanced neoantigen quality^24,27,28^.

In parallel, WES-derived TMB was significantly higher in Cohort A compared to Cohort C (median: 2.8 mutations/Mb [IQR: 2.2–4.0] vs 1.25 [IQR: 0.50–1.90], *p*=0.035; (**Supplementary Fig. 5a**). IMPACT-HRD scores were also significantly higher in Cohort A compared to Cohort C [median: 47 [IQR: 22–61] vs 24 [IQR: 12–37] (**Supplement Fig. 5a,c**). These genomic features were accompanied by greater CD3+ TIL density on H&E in Cohort A than in Cohort C (median: 3.5 [IQR: 3–4] vs 2 [IQR: 2–3], p=0.035) (**Supplementary Fig. 5c**). Across all samples, IMPACT-HRD score positively correlated with predicted neoantigen burden (R=0.48, *p*=0.008; **Supplementary Fig. 6a**).

#### Neoantigen Burden Correlates with HRD but Not with CD8+T Cell Infiltration

Using multiplex immunofluorescence (mIF), we quantified immune infiltration across samples with available neoantigen and IMPACT_HRD score. While CD3+ T cell and inferred CD4+ T cell (CD3+CD8-) infiltration showed modest positive trends with neoantigen burden (R=0.31, *p*=0.31 and R=0.30, *p*=0.23, respectively), CD8+T cells showed no correlation (*p*=0.95). CD68+ macrophage infiltration trended inversely with IMPACT-HRD score (R=−0.41, *p*=0.089). PD-L1 expression and T cell-to-macrophage ratios did not significantly correlate with IMPACT-HRD score or neoantigen burdens (**Supplementary Fig. 6**).

## Discussion

Pancreatic cancer (PC) remains one of the most challenging malignancies to treat, with limited efficacy from immune checkpoint blockade (ICB) in part due to poor immunogenicity and a highly immunosuppressive tumor microenvironment (TME)^29,30^. The POLAR phase II trial was designed to evaluate whether combining a PARP inhibitor with PD-1 blockade during a chemotherapy-free maintenance window could yield durable benefit in patients with metastatic PC after response to platinum-based chemotherapy. Although the POLAR trial did not meet its prespecified co-primary endpoints (ORR of 43% and a 6-month PFS of 77% in *BRCA1/2/PALB2*-mutant Cohort A), these thresholds were intentionally set high in early trial planning to guide feasibility and signal strength in a rare population. Importantly, patients enrolled in POLAR represented a highly selected subset with favorable disease biology, particularly those in Cohort A who exhiobited strong platinum sensitivity. In many cases, RECIST evaluability was anticipated limitation at the time of trial enrollment due to marked radiographic responses. In retrospect, they may have been overly ambitious for a non-registrational study. Nonetheless, a clinically meaningful signal was observed, with durable responses and prolonged survival in a subset of patients, supporting further investigation of this therapeutic approach.

In Cohort A, the observed ORR of 35% (95% CI: 15–59), with a 6-month PFS rate of 64% (95% CI: 49–82), and a 2-year OS rate of 56%, compares favorably to historical benchmarks such as the POLO (Pancreas OLaparib Ongoing) trial (olaparib monotherapy: ORR 23%, 6-month PFS rate 53% and 2-year OS rate 37%)^31^. Although the olaparib arm in the POLO trial demonstrated a PFS benefit, no OS improvement was observed (hazard ratio 0.83 [95% CI: 0.56–1.22], *p* = 0.3487), leading to ongoing debate about the role of olaparib monotherapy in PC maintenance^5,6,31^. POLAR was designed to test a new therapeutic paradigm by incorporating pembrolizumab, an anti-PD-1 ICB – a strategy supported by retrospective clinical data and preclinical evidence that DNA damage repair (DDR) deficiency may activate innate immune sensing pathways such as cGAS-STING, rendering tumors more susceptible to immunotherapy^32,33^.

Across various cancers, PARP inhibitors combined with immune check blockade (PARPi-ICB) have demonstrated variable activity. In the tumor-agnostic, phase 2 KEYLYNK-007 trial (N=322), durable responses to pembrolizumab plus olaparib were reported among patients with homologous repair (HRR) mutations (e.g., *BRCA1*/*2*, *PALB2*, *ATM*, *RAD51D*)^34^. Among 15 participants with pancreatic cancer (PC) and *BRCA1*/*2* mutations, 4 achieved partial responses, which was remarkable given the inclusion of heavily pretreated and platinum-refractory cases. In contrast, participants in the POLAR trial were enrolled with platinum-sensitive disease, a so-called “molecularly” residual disease (mRD) state, a context increasingly recognized as optimal for immune engagement in PC, and as necessitated in adjuvant cancer vaccine trials^15–17^. Additional PARPi-ICB trials in ovarian, breast, endometrial, and lung cancers reported modest activity, particularly in HRD-enriched tumors^35–37^. However, optimal selection criteria – including mutational types, timing, and TME context – remain unresolved. Cross-trial evidence suggests that greatest benefit is achieved in core HRD (biallelic *BRCA1/2 or PALB2* loss) tumors or HRD-signature-high setting (e.g., COSMIC SBS3, GIS, HRDetect, IMPACT-HRD) in immune-permissive contexts^19,20,22,38,39^.

The biomarker-integrated design in POLAR enabled comprehensive translational analyses to be conducted. HRD tumors (Cohort A) demonstrated significantly higher WES-TMB, IMPACT-HRD, indel, and frameshift indel burden compared to HRP tumors (Cohort C), consistent with higher genomic instability (**Supplementary Fig. 6a-c**). These molecular features trended with higher predicted neoantigen burden and increased CD3+T cell infiltration, suggesting enhanced immunogenicity. However, the link between antigenicity and immune infiltration was incomplete. Some HRD tumors with high neoantigen burden showed limited T cell infiltration, suggestion that additional barriers of impaired antigen presentation or immune exclusion may restrict immune engagement despite genomic instability (**Supplementary Fig. 7b,d**).

Immune exclusion in PC is multifactorial. Our findings, consistent with prior reports, indicate that neoantigen presence alone does not guarantee CD8+ T cell infiltration^40^. Mechanisms such as CAF-mediated FoxP3+ regulatory T cells (Treg) recruitment and structured exclusionary niches^41^ as well as oncogenic *KRAS*^G12D^-driven CD11b+ myeloid cell enrichment^42,43^, likely suppress effector immune cell entry. In this study, HRD tumors had elevated CD3+ TILs although no clear correlation between neoantigen burden and CD8+T cell infiltration was observed, reinforcing the role of additional suppressive axes beyond antigen presentation. These results suggest that while neoantigen recognition is necessary, it is not sufficient for robust immune engagement in PC.

Fifteen participants (N=13 from Cohort A and N=2 from Cohort B) remained on therapy beyond one year (PFS > 1 year), while only a few in Cohort C achieved sustained disease control. This disparate outcome suggests that individual HRD or ncHRD mutations may influence therapeutic sensitivity and underscores the importance of molecular stratification. Moreover, exploratory analysis of cfDNA dynamics suggested that molecular residual disease (mRD) negativity, defined by undetectable or near-undetectable mean VAF, may be associated with durable benefit, highlighting a possible role of mRD as a clinical indicator for future immunotherapy trials.

Our study has limitations. The single-center, multi-cohort parallel cohort non-randomized, single-arm design and modest sample size limit generalizability. High sample quality control failure, while expected in PC, limited complete multiomic profiling. Strengths include complete participant follow-up, prospective biospecimen collection and comprehensive integrated genomic and immunologic analyses providing notable insights for the clinical observations.

Looking ahead, rational combination strategies are needed to overcome barriers to effective immune response in PC. Beyond PARP, the DNA damage response (DDR) therapeutic landscape is rapidly evolving with next-generation targets such as *POLQ, ATR, WEE1, CHK1*, PARG, and tankyrase inhibitors in clinical development^44^. Integration of DDR-targeted agents with TME-modulating therapies, such as *KRAS* inhibitors, myeloid/CD11b modulators, or CAF-directed approaches, may enhance T cell infiltration and anti-tumor response. Spatial analysis, TCR tracking, mutational signatures associated with neoantigens, and functional assays of these interactions are ongoing for the POLAR trial. The POLAR trial offers a model for biomarker-integrated precision immunotherapy studies and underscores the critical role of HRD, neoantigen, and immune contexture in shaping therapeutic outcomes in pancreatic cancer.

## Conclusion

In conclusion, while not meeting the prespecified primary endpoints, the POLAR trial demonstrates that a biomarker-guided maintenance approach combining PARP inhibition with anti-PD-1 immune checkpoint blockade is feasible and yields durable clinical benefit in a notable subset of patients with PC. In Cohort A, comprised of patients with core HRD (*BRCA1/2* or *PALB2* mutations), we observed high tumor response rates, durable disease control, and encouraging long-term survival. These findings support a model in which DDR-driven genomic instability promotes tumor immunogenicity, acknowledging immune engagement is ultimately shaped by additional suppressive features within the tumor microenvironment. These results provide a rationale for ongoing randomized studies (e.g., SWOG S2001, NCT04548752) and future precision immunotherapy trials that integrate DDR status, neoantigen quality, and TME remodeling to improve outcomes in patients with pancreatic cancer.

## Methods

### Study Design

This was a single-institution, open-label, non-randomized Phase 2 trial conducted at Memorial Sloan Kettering Cancer Center (NCT04666740) (Supplementary 1. Protocol). Participants with metastatic pancreatic cancer (PC), aged ≥18 years and with ECOG performance status 0–1, were enrolled into three biomarker-defined cohorts: Cohort A: *BRCA1/2* or *PALB2*-mutant tumors (core HRD), Cohort B: tumors harboring non-core HRR gene mutations, Cohort C: homologous recombination–proficient (HRP) tumors with durable platinum sensitivity.

Eligible participants had histologically confirmed pancreatic adenocarcinoma (including adenosquamous or acinar subtypes), completed platinum-based induction therapy, and had no disease progression prior to enrollment. Cohort A and B for 4 months and Cohort C for 6 months. Exclusion criteria included >2 prior lines of systemic therapy, prior PARP inhibitor (PARPi) or immune checkpoint blockade (ICB), or radiographic progression before trial entry.

Participants received maintenance pembrolizumab (anti–PD-1 antibody) and olaparib (PARPi). The dual primary endpoints were: 6-month progression-free survival (PFS) rate ≥77%, and objective response rate (ORR) ≥43%, both evaluated in Cohort A using RECIST v1.1. Secondary endpoints included disease control rate (DCR), median PFS, overall survival (OS), biochemical response (CA19–9 and CEA), safety (CTCAE v5.0), and best overall response.

A two-stage design was employed in Cohort A for dual co-primary endpoints (see **Supplementary Methods 1**).

### Treatment and Follow-up

All participants received maintenance olaparib (300 mg BID) and pembrolizumab (every 3 weeks for 6 months, then every 6 weeks until disease progression or unacceptable toxicity). Select patients were allowed to continue beyond radiographic progression if deriving clinical benefit. Enrollment occurred from December 28, 2020, to February 20, 2024 (data cutoff: June 25, 2025).

### Exploratory Correlative Endpoints

Included: tumor WES, MSK-IMPACT genomic profiling, cfDNA monitoring via MSK-ACCESS, IMPACT-HRD score, neoantigen prediction, multiplex immunofluorescence (mIF), and hematoxylin and eosin (H&E) imaging.

### Radiographic Response Assessment

Baseline CT imaging was performed within 28 days of enrollment. Imaging occurred every 9 weeks (±7 days) through Cycle 10, then every 12 weeks. RECIST v1.1 criteria were applied by central blinded radiology review using Mint Lesion^™^ (Mint Medical), with structured lesion annotation and longitudinal tracking. Participants receiving therapy >2 years could undergo imaging every 3–6 months. Patients were permitted to remain on treatment beyond progression with PI discretion if clinical benefit was observed.

### Research Biospecimen Collection

Image-guided tumor biopsies were obtained at three timepoints: baseline (T1), on-treatment (Cycle 2–3, T2), and post-progression (T3), where feasible. Samples were formalin-fixed paraffin embedded (FFPE) or snap-frozen for WES, and mIF. Peripheral blood was collected at six timepoints for PBMC and cfDNA analysis (baseline, on-treatment, C6, C12, C18, and progression).

### Whole exome sequencing (WES)

Tumor-normal paired WES data were processed using TEMPO pipeline (https://github.com/mskcc/tempo), Nextflow-based pipeline developed by the Center for Molecular Oncology at MSKCC for both WES and WGS data. The TEMPO pipeline performs alignment, quality control, somatic and germline variant detection, and generates annotated output suitable for downstream analysis. Somatic mutations (single nucleotide variants, insertions, and deletions) were called in tumor-normal pairs using MuTect2 (v4.1.0.0) and Strelka2 (v2.9.10)^45^. Variants were annotated and filtered for recurrent artifacts and false positives using methods previously described^46^.

### Genomic Instability and IMPACT-HRD Score

IMPACT-HRD quantifies genomic scars associated with homologous recombination deficiency (HRD) by analyzing allele-specific copy-number alterations determined with the FACETS algorithm^47^ (version 0.5.14) and computing those genomic scars with the impact-hrd package (https://github.com/danielmuldoon/impact-hrd/). All IMPACT-HRD assessments were completed using R Version 4.1.2. In particular, three metrics are evaluated: number of telomeric allelic imbalances (NtAI), large-scale transitions (LST), and losses of heterozygosity (HRD-LOH). The overall HRD phenotype is defined as the unweighted sum of these three metrics (HRD-sum), with additional consideration given to whole-genome duplication (WGD) status.

### Neoantigen Prediction

Somatic mutations from WES or MSK-IMPACT were analyzed using NetMHCpan v4.0 to predict MHC class I binding affinity. 8–11mer peptides with predicted binding affinity ≤500 nM were considered candidate neoantigens. To account for both quantity and binding strength, a **weighted neoantigen burden** was computed per sample by summing the inverse binding affinity (1 / IC50) across all retained peptides. This score emphasizes the contribution of strong binders and was used for sample ranking and correlation analyses.

### Cell-Free DNA: MSK-ACCESS

The MSK-ACCESS liquid biopsy test was employed to study circulating tumor DNA in the plasma. An in-depth description of this assay has been published earlier^48^. This test uses hybridization capture and deep sequencing to discover low-frequency somatic changes and pinpoint different kinds of genomic irregularities, such as single nucleotide polymorphisms, base insertions or deletions, and copy number alterations. The test scrutinizes selected exons and introns from 147 genes, which have been previously identified to be defective in human cancers. It’s important to note that this assay can detect variant allele frequencies (VAFs) as low as 0.1%. The technique uses matched white blood cell sequencing to isolate and filter out germline results from the ctDNA findings and can distinguish mutations linked to clonal hematopoiesis^49^. The New York State Department of Health sanctioned this test for clinical application on May 31, 2019.

56 samples from 31 patients were studied with the MSK-ACCESS and the results were juxtaposed with those derived from tumor that underwent MSK-IMPACT (29 patients)^50^. The variants (SNV and INDELs) resulting from the procedure were manually examined using the Integrated Genomics Viewer (v0.4.0). For 29 patients, where samples were profiled by both MSK-ACCESS and MSK-IMPACT, variants were genotyped across the shared region between the two assays utilizing GetBaseCounMultiSample (v1.2.5). VAF was computed from somatic, non-CH mutations. Longitudinal VAF dynamics were analyzed when paired timepoints are available.

### Multiplex Immunofluorescence (mIF)

mIF was performed on FFPE tissue from 21 patients using a validated panel: CD3, CD8, CD68, PD-L1, and DAPI. Imaging was conducted on the Vectra Polaris platform. Qupath v0.5.1 was used for automated quantification. Tumor and stromal compartments were annotated by a pathologist. Marker-positive cells were normalized to stromal cell count. Correlation between mIF markers, TMB, and cohort was computed using ggpubr in R (v4.2.2).

Automated multiplex immunofluorescence was done using a Leica Bond RX staining system. FFPE tissues from 21 patients were sectioned at 5 μm and baked at 58 °C for 1 hr before loading in the Leica Bond and stained using the following protocol: Samples were dewaxed at 72 °C and treated with ER2 epitope retrieval solution (Leica Biosystems, AR9640) for 20 min at 95°C. A 5-plex PD-L1/CD8/CD68/CD20/CD3 panel was applied sequentially using the following antibodies: PD-L1 (Cell Systems Technologies, clone E1L3N, 1:2000 dilution) CD8 (Roche Ventana, clone SP57, 1:200 dilution); CD20 (DAKO, clone L26, 1:2000 dilution); CD68 (DAKO, clone KP1, 1:200 dilution); CD3 (Roche Ventana, clone 2GV6, 1:2000 dilution). After incubation with an antibody for 1hr, Leica Bond Polymer anti-rabbit HRP was applied, followed by the relevant fluorophore (tyramide conjugated Alexa Fluor dye 488 or 647 (Life Technologies B40953 and B40958) or tyramide conjugated CF dye 430, 543 or 594 (Biotium 96053, 92172, 92174)) for signal amplification. Epitope retrieval was repeated between rounds of staining to denature antibodies before addition of the next primary antibody. After IF staining, samples were counterstained with 5 μg/ml DAPI (Sigma-Aldrich), rinsed briefly in PBS and mounted in Mowiol-488 (Calbiochem). Slides were scanned on a Pannoramic MIDI II whole-slide scanning fluorescence microscope (3DHistech) with a 20x/0.8 NA air objective and visualized in QuPath 0.4.2^51^.

### Statistical Methods

Summary statistics were used to describe baseline demographic, biomarkers, IMPACT genomic, and safety profiles of enrolled patients according to cohorts.

Overall survival (OS) and progression free survival (PFS) were calculated from date of treatment initiation until date of first disease progression or death (for PFS) or to date of death (for OS). OS and PFS were estimated using Kaplan-Meier methods and reported separately for the 3 cohorts. Disease control rate (DCR) was defined as the percentage of patients who achieved complete response, partial response and stable disease per RECIST version 1.1. Duration of response was calculated among responders from the date of best response until date of disease progression and estimated using Kaplan-Meier methods.

Change in mean variant allele frequency (VAF) from baseline (T1) to on-treatment (T2, approximately 3 weeks) was summarized for 3 cohorts together and compared between dichotomized PFS time points (shorter than or equal to 6 months vs longer than 6 months) using Wilcoxon Rank-Sum test. Finally, exploratory endpoints were summarized using descriptive summary statistics and compared between cohorts using Wilcoxon-rank sum test. Correlation between immune cell infiltration with IMPACT-HRD score and neoantigen burden was evaluated using Pearson’s correlation coefficient. All analyses were performed using R (version 4.3.2). All *P*-values were two-sided and *p*-values of <0.05 were considered to indicate statistical significance.

## Supplementary Material

Table 1. Demographic and baseline clinical characteristics of POLAR trial participants by biomarker-defined cohort.

Baseline clinical characteristics are presented for all 63 participants enrolled in the POLAR trial, stratified by biomarker-defined cohorts: Cohort A (core HRD; *N*=33), Cohort B (non-core HRD [ncHRD]; *N*=15), and Cohort C (HRD-negative, platinum-sensitive; *N*=15). Variables include age, sex, race, initial stage at diagnosis, histologic subtype, prior surgery, platinum agent received during induction therapy. ECOG performance status at trial entry is also shown.

**Abbreviations:** UNK, unknown; HRD, homologous recombination deficiency; ncHRD, non-core HRD; PR, partial response; SD, stable disease; ECOG, Eastern Cooperative Oncology Group performance status; IQR, interquartile range.

Table 2. Clinical and molecular characteristics of POLAR trial participants stratified by biomarker-defined cohort.

This table summarizes key clinical responses, genomic biomarkers, and immune features across the three POLAR cohorts: (A) HRD, (B) non-core HRD (ncHRD), and (C) HRD-negative platinum-sensitive (HRP). Parameters include family history of pancreatic cancer, and best overall response (BOR) by RECIST (CR, PR, SD, POD). Median tumor mutational burden (TMB), genomic instability score (GIS), predicted neoantigen load, and tumor-infiltrating lymphocyte (TIL) density at baseline (T01) are reported. CA19–9, CEA, and neutrophil-to-lymphocyte ratio (NLR) at T1 are also shown.

**Abbreviations**: BOR, best overall response; CR, complete response; PR, partial response; SD, stable disease; POD, progressive disease; HRD, homologous recombination deficiency; ncHRD, non-core HRD; HRP, homologous recombination proficient; TMB, tumor mutational burden; GIS, genomic instability score; TIL, tumor-infiltrating lymphocytes; NLR, neutrophil-to-lymphocyte ratio; CA19–9, carbohydrate antigen 19–9; CEA, carcinoembryonic antigen; IQR, interquartile range.

Table 3. Treatment-related adverse events (TRAEs) and immune-related adverse events (irAEs).

Grade 2 and Grade 3 adverse events attributed to olaparib (top) and pembrolizumab (bottom) are shown. For olaparib, Grade 3 anemia occurred in 10 patients (100% of Grade 3 events), while Grade 2 events were reported in 15 patients with anemia, fatigue, and nausea as the most common. For pembrolizumab, Grade 3 events occurred in 3 patients, including diarrhea, pneumonitis, and hyperglycemia. Immune-related adverse events (irAEs) attributed to pembrolizumab included pneumonitis, colitis, hyperthyroidism, and hyperglycemia. Counts reflect number of events and the proportion of affected patients. No Grade 4 or 5 treatment-related events were observed.

**Abbreviations:** TRAE, treatment-related adverse event; irAE, immune-related adverse event.

Supplementary Method 1. Protocol IRB 20–481

Supplementary Figure 2. Cohort A subgroup analysis of PFS and OS by HRD mutations

(a) Distribution of HRD mutations in Cohort A (N = 33), showing the proportion of *BRCA1*, *BRCA2*, and *PALB2* mutations.

(b) Median PFS in months by mutation type with 95% confidence intervals (CI).

(c) Kaplan–Meier curves of PFS stratified by *BRCA1* (blue), *BRCA2* (red), and *PALB2* (purple).

(d) Median OS in months and landmark survival rates at 12 and 24 months by mutation type.

(e) Kaplan–Meier curves of OS stratified by *BRCA1* (blue), *BRCA2* (red), and *PALB2* (purple).

Censoring is indicated by tick marks. Survival estimates are based on investigator-assessed outcomes. Numbers at risk and number of events are displayed as tick in each Kaplan–Meier plot.

**Abbreviations:** OS, overall survival; PFS, progression-free survival

Supplementary Figure 3. Cohort B subgroup analysis of PFS and OS by *ATM* mutation status.

(a) Distribution of ATM or other ncHRD mutations in Cohort B (N = 15), showing 60% (9/15) of patients harbored ATM mutations.

(b) Median PFS by *ATM* mutation status with 95% confidence intervals (CI).

(c) Kaplan–Meier curve of PFS of participants stratified by *ATM* (1, red) or other ncHRD (0, blue).

(d) Median OS by *ATM* mutation status with 95% CI.

(e) Kaplan–Meier curve of OS of participants stratified by *ATM* (1, red) or other ncHRD (0, blue). Numbers at risk and number of events are shown below each curve. Survival outcomes were investigator-assessed. No statistically significant differences were observed, but trends are noted.

**Abbreviations:** OS, overall survival; PFS, progression-free survival

**Supplementary Table 1**. Cohort A and B subgroup analysis.

Supplementary Figure 4. Plasma-based mutational profiling using MSK-ACCESS emphasizes molecular minimal disease dynamics during POLAR maintenance.

(a) Density plot showing the distribution of mean variant allele frequency (VAF) at baseline (T1) and on-treatment (T2) timepoints. Most samples exhibited very low VAF at T1, consistent with molecular residual disease (mRD) at T1.

(b) Scatterplot of cfDNA VAF change (T2 – T1) versus best RECIST (% tumor change). A red vertical line marks the radiographic response (RECIST v1.1) at 0%.

(c) Paired line plot showing cfDNA mean VAF dynamics over 6 weeks in 30 representative plasma pairs across cohorts A–C, stratified by PFS ≤ 6 months (purple) and > 6 months (red). A trend toward lower VAF change was observed in long-term responders (p = 0.063).

(d) Summary table of select participants with durable PFS (> 36 months), highlighting their HRD status, neoantigens, PFS, treatment details, and cfDNA metrics. Four of the five patients had undetectable or near-undetectable cfDNA at both timepoints, suggesting a link between mRD negativity and durable benefit.

(e) Oncoprint summarizing somatic mutations detected by MSK-ACCESS in 56 evaluable samples. Only genes altered in ≥2 samples are shown. Mutation type, timepoint (T1 vs T2), OncoKB level, and gender are annotated.

**Abbreviations:** cfDNA, cell-free DNA; VAF, variant allele frequency; PFS, progression-free survival; mRD, molecular residual disease; TMB, tumor mutational burden; T1, baseline; T2, 6-week on-treatment; OncoKB, Onco Knowledge Base.

Supplementary Figure 5. Immunogenomic context of tumors from POLAR.

(a) **Baseline genomic characteristics** from WES (N=35) stratified by POLAR cohort (A: HRD, B: ncHRD, C: HRP): (i) Tumor purity, (ii) WES-derived tumor mutational burden (WES_TMB) was significantly higher in Cohort A (HRD) *p* < 0.05, (iii) ploidy, and (iv) fraction genome altered (FGA).

No statistically significant differences were observed across cohorts.

(b) **Mutation types and neoantigen burden**:

(i) Number of nsSNVs, (ii) total indel burden, (iii) frameshift indel burden, and (iv) predicted neoantigen burden. Indel and frameshift indel burden were significantly higher in Cohort A (HRD). Statistical comparisons were performed using Wilcoxon rank-sum tests. Significance is indicated by *p* values (p < 0.01).

(c) **Baseline genomic and immune features** across platforms: IMPACT HRD score (N=35), IMPACT TMB (N=42), neoantigen burden from WES (N=35), TIL density from H&E (N=38), and baseline neutrophil-to-lymphocyte ratio (NLR) from blood (N=63).

No significant differences were observed between cohorts in these parameters.

Each dot represents an individual patient. Boxes indicate median and interquartile range; whiskers represent 1.5× IQR. Cohorts: A (purple), B (teal), C (navy). Statistical significance was assessed by two-sided Wilcoxon test and annotated as: ns (not significant), *p* < 0.05, *p* < 0.01.

**Abbreviations:** WES, whole exome sequencing; HRD, homologous recombination deficiency; ncHRD, non-core homologous recombination deficiency; nsSNVs, nonsynonymous single-nucelotide variants; OS, overall survival; PFS, progression-free survival

Supplementary Figure 6. Correlation of immune cell infiltration with IMPACT-HRD score and neoantigen burden in the POLAR cohort (N = 27).

Scatter plots display Pearson correlation coefficients (R) and associated p-values between genomic features (IMPACT-HRD score or neoantigen burden) and immune cell infiltration metrics, assessed via multiplex immunofluorescence (mIF). Linear regression lines are shown with 95% confidence intervals (gray shading). Each dot represents a patient sample, color-palette by Cohort A (HRD, purple), B (ncHRD, teal), and C (HRP, navy).

(a) Neoantigen burden significantly correlates with IMPACT-HRD score (R = 0.48, p = 0.008).

(b) CD3^+^ T cell infiltration shows a modest positive trend with neoantigen burden (R = 0.31, p = 0.2).

(c) CD68+ macrophage infiltration trends inversely with IMPACT-HRD score (R = −0.41, p = 0.089).

(d) CD4^+^ T cell infiltration, inferred as CD3^+^CD8^−^, shows a modest positive trend with neoantigen burden (R = 0.30, p = 0.23).

CD4^+^ T cells were not directly stained and were approximated as CD3^+^CD8^−^ cells. All correlations were evaluated using Pearson’s method.

**Abbreviations:** WES, whole exome sequencing; HRD, homologous recombination deficiency; ncHRD, non-core HRD; mIF, multiplex immunofluorescence; TMB, tumor mutational burden; OS, overall survival; PFS, progression-free survival.

Supplementary Files

This is a list of supplementary files associated with this preprint. Click to download.

- 6.Supplementary1MSKIRB20481POLARProtocol.pdf
- 7.SupplementaryTable1HRDCohortABSubgroupAnalysisTable.xlsx
- Tables.pdf
- POLARmanuscript.r
- SupplementaryInformation.pdf
